# Breathlessness and exercise with virtual reality system in long-post-coronavirus disease 2019 patients

**DOI:** 10.3389/fpubh.2023.1115393

**Published:** 2023-02-23

**Authors:** Vasileios T. Stavrou, George D. Vavougios, Periklis Kalogiannis, Konstantinos Tachoulas, Evlalia Touloudi, Kyriaki Astara, Dimitrios S. Mysiris, Glykeria Tsirimona, Eirini Papayianni, Stylianos Boutlas, Mary Hassandra, Zoe Daniil, Yannis Theodorakis, Konstantinos I. Gourgoulianis

**Affiliations:** ^1^Laboratory of Cardio-Pulmonary Testing and Pulmonary Rehabilitation, Respiratory Medicine Department, Faculty of Medicine, University of Thessaly, Larissa, Greece; ^2^Department of Neurology, Faculty of Medicine, University of Cyprus, Lefkosia, Cyprus; ^3^Department of Physical Education and Sport Sciences, University of Thessaly, Trikala, Greece; ^4^Department of Neurology, 417 Army Equity Fund Hospital, Medical Institution Military Shareholder Fund (NIMTS), Athens, Greece; ^5^Faculty of Medicine, University of Thessaly, Larissa, Greece; ^6^Department of Respiratory Medicine, School of Health Sciences, University of Thessaly, Larissa, Greece

**Keywords:** dyspnea, virtual reality system, cognitive impairment, fitness indicators, long-post-COVID-19

## Abstract

Long-post-coronavirus disease-2019 (COVID-19) patients tend to claim residual symptomatology from various systems, most importantly the respiratory and central nervous systems. Breathlessness and brain fog are the main complaints. The pulmonary function pattern is consistent with restrictive defects, which, in most cases, are self-resolved, while the cognitive profile may be impaired. Rehabilitation is an ongoing field for holistic management of long-post-COVID-19 patients. Virtual reality (VR) applications may represent an innovative implementation of rehabilitation. We aimed to investigate the effect of exercise with and without the VR system and to assess further breathlessness and functional fitness indicators in long-post-COVID-19 patients with mild cognitive impairment after self-selected exercise duration using the VR system. Twenty long-post-COVID-19 patients were enrolled in our study (age: 53.9 ± 9.1 years, male: 80%, body mass index: 28.1 ± 3.1 kg/m^2^). Participants' anthropometric data were recorded, and they underwent pulmonary functional test evaluation as well as sleep quality and cognitive assessment. The participants randomly exercised with and without a VR system (VR vs. no-VR) and, later, self-selected the exercise duration using the VR system. The results showed that exercise with VR resulted in a lower dyspnea score than exercise without VR. In conclusion, VR applications seem to be an attractive and safe tool for implementing rehabilitation. They can enhance performance during exercise and benefit patients with both respiratory and cognitive symptoms.

## 1. Introduction

Dyspnea is characterized by a subjective experience of breathing discomfort that comprises qualitatively distinct sensations that vary in intensity while deriving from interactions among multiple physiological, psychological, social, and environmental factors and may induce secondary physiological and behavioral responses (1). During exercise, obstructive and restrictive lung disease, dyspnea, and exercise intolerance can be attributed to impaired gas exchange, exaggerated ventilatory responses to exercise, dynamic hyperinflation, and/or elevated pulmonary vascular pressures (2). In long-post-coronavirus disease-2019 (COVID-19) patients, pulmonary function patterns are consistent with restrictive defects, which normalize over time (3), while pulmonary function tests may be insufficient to evaluate abnormal dyspnea after COVID-19 (4). In contrast, persistent dyspnea after COVID-19 seems unrelated to overt cardiopulmonary impairment or exercise intolerance (5).

Virtual reality (VR) applications represent a novel and efficient approach to rehabilitation (6). As the pandemic has limited rehabilitation options and access to healthcare, VR has been used successfully in both in-patient and out-patient settings (7, 8). Notably, VR rehabilitation does not only include physical and respiratory regimens but also cognitive regimens (9). Many studies support the fact that exercise in a VR environment may have positive effects on psychological, physiological, and rehabilitation outcomes compared with traditional exercise (10). It could be attractive to many people interested in gaming and entertainment as it could involve all senses and help provide personalized training conditions by adjusting the intensity and difficulty level of someone's needs (11). Exercise combined with VR is considered an effective method for the treatment and prevention of many chronic diseases. A VR environment can enhance the beneficial effects of exercise and increase adherence to training programs. Exercise in a VR environment, combining physical and cognitive tasks, could increase neuroplasticity and lead to improved problem-solving ability and sensorimotor integration (12). Moreover, VR exercise is not only considered a new approach in rehabilitation but also a new way of promoting and preventing non-communicable diseases. Many studies have shown that exercise in VR environments can increase the training frequency and strength of physical performance (13).

Information in the literature on the impact of VR exercise in long-post-COVID-19 patients and its impact on fitness indicators, dyspnea, and leg fatigue is insufficient (14). Therefore, our study aimed to investigate the effect of exercise with and without a VR system (VR vs. no-VR) and assess, breathlessness and indicators of functional fitness in long-post-COVID-19 patients with mild cognitive impairment after self-selected exercise duration using the VR system (SSE-VR). The VR training system was selected for use in patients because it is safe, easily controlled, and attractive for exercise (15).

## 2. Methods

### 2.1. Participants

**Figure 1:**
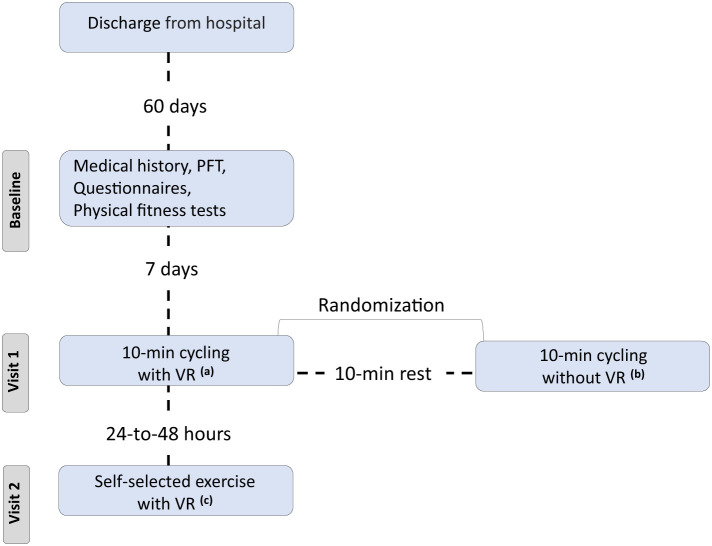
Flow chart. PFT, pulmonary functional test; VR, virtual reality system. ^a^Exercise using the VR system and 10-min duration, ^b^Exercise without using the VR system and 10-min duration, ^c^Exercise using the VR system, and each patient selected the duration of session.

Twenty consecutive COVID-19 participants, previously hospitalized at the University Hospital of Larissa, Greece (Figure 1) from November, 2021 to January, 2022 (Delta variant), were included in our study (Table 1). All patients were recruited 2 months post discharge.

**Table 1:** Long-post-COVID-19 patient characteristics.

		**Long-post-COVID-19**
Age	years	53.9 ± 9.1
Sex (F)	*n* (%)	4 (20%)
Length of stay	days	5.7 ± 2.1
Body mass index	kg/m^2^	28.1 ± 3.1
Body fat	%	29.0 ± 5.1
Muscle mass	%	31.7 ± 2.5
Visceral fat	score	11.9 ± 2.8
Total body water	%	51.0 ± 3.6
Waist–hip ratio	score	0.9 ± 0.8
Δchest	cm	7.1 ± 1.3
6-minute walk distance	m	584.5 ± 86.2
30-s Sit-to-Stand test	rep	12.5 ± 2.7
Handgrip strength test	kg	38.4 ± 5.6
d-ROMs	U. carr.	316.1 ± 29.1
FEV_1_	% of predicted	102.0 ± 11.8
FVC	% of predicted	97.5 ± 12.9
PEF	% of predicted	112.0 ± 11.3
DLCO(SB)	% of predicted	78.9 ± 19.8
PSQI	score	4.9 ± 3.0
STOP-Bang	score	3.1 ± 1.4
WAI	score	30.8 ± 11.3
MoCA	score	24.3 ± 1.5

Data are expressed as percent, mean ± standard deviation.

DLCO(SB), single-breath diffusing capacity of the lung for CO; d-ROMs, determination of reactive oxygen metabolites' levels; F, female; FEV_1_, forced expiratory volume in 1st sec; FVC, forced vital capacity; MoCA, Montreal Cognitive Assessment questionnaire; PEF, peak expiratory flow; PSQI, Pittsburg sleep quality index questionnaire; PSQI, Pittsburgh sleep quality index questionnaire; WAI, work ability index questionnaire; Δchest, chest circumference difference between maximal inhalation and exhalation.

Inclusion criteria were at least 2 months post discharge, without fever for a 48-h period, stable, without supplemental O_2_, age ≥ 20 and ≤70 years, without absolute (unstable angina during the previous month and myocardial infarction during the previous month) and relative (resting heart rate >120 bpm, systolic blood pressure >180 mmHg, and diastolic blood pressure >100 mmHg) contraindications for the 6-min walk test (6MWT), body mass index <35 kg/m^2^, comorbidity free (i.e., musculoskeletal disability, cardiorespiratory diseases, etc.), color blindness, 6MWT ≥85% of predicted (16, 17) and Montreal Cognitive Assessment questionnaire score, <26.

Permission for the study was granted by the institution's ethics committee (approval number: 1829; October 13, 2021). The confidentiality of personal data was assured with Regulation (EU) 2016/679 (General Data Protection Regulation). The participants were informed orally of the procedure, provided written information, and signed the consent form. Additionally, they were encouraged to ask questions and receive complete and detailed answers.

### 2.2. Data collected

The anthropometric characteristics were recorded as described by Stavrou et al. (16). Tanita MC-980 (Arlington Heights, IL, USA) was used for body composition assessment. We performed the 6MWT, as described in the ATS guidelines, to assess the functional status of the patients (17). The parameters O_2_ saturation (SpO_2_), heart rate (HR) (Nonin 9590 Onyx Vantage, USA), blood pressure (Mac, Tokyo, Japan), and self-assessed lower limb fatigue and dyspnea (*via* Borg Scale CR-10) (18) were recorded at predetermined time-points of the 6MWT (16). The handgrip strength test was performed using an electronic dynamometer (Camry EH 101, South El Monte, CA, USA) (19). Patients were asked to perform as many repetitions as possible at a self-regulated pace (safe and comfortable) from a sitting-to-standing position while the arms were crossed at the shoulders so as not to use them as support to assess lower limb strength (30-s Sit-to-Stand test) (20). Blood sampling of 10 mL peripheral venous blood for the determination of reactive oxygen metabolites (d-ROMs test, free radical analytical system, FRAS5, Parma, Italy) was performed 20 min before physical fitness tests (21). Pulmonary function tests were performed according to the ATS/ERS guidelines (22) in the sitting position using a MasterScreen-CPX pneumotachograph (VIASYS HealthCare, Germany). Prior to physical fitness tests, all patients answered questionnaires to measure the quality and patterns of sleep using the Pittsburgh Sleep Quality Index (PSQI) (23), cognitive impairment was assessed using the Montreal Cognitive Assessment (MoCA) (24), STOP-Bang for stratification for obstructive sleep apnea risk (25), and (iv) work ability index (WAI) to investigate the ability to return to work without restrictions (26).

A stationary seated bike (Toorx, Chrono Line, BRX R 300) with bluetooth capabilities was used for the measurements. It was connected to the VR application, the Meta Quest 2 (Facebook Technologies, LCC, Hacker Way, Menlo Park, CA, USA) device headset and controllers, and a computer (27). This VR training system is called VRADA (VR exercise App for Dementia and Alzheimer's patients) version 4.1 and has been developed by ORAMA-VR and Biomechanical Solutions Engineering based on interviews with older people with mild cognitive impairment. The application of the VR training system includes cognitive exercises with simple math calculations and requests users to observe and count animals that appear in their VR to enhance cognitive health and motivational techniques to address the issue of low motivation for exercise. The system gives an opportunity for each participant to choose their exercise duration, landscape in which they will cycle (forest, beach, or snowy landscape), motivating words that they want to hear during their performance (“Calmly,” “I can,” “I will do it well,” “Very nice,” or no words) and the music to enjoy while cycling. VR controllers with raycast were used as a selection mechanism that allows the user to select an answer by pointing the ray at the button and pressing the trigger button at the controller. Moreover, participants received feedback during their performance, such as indications about cycling time, distance, and speed, and could self-monitor their performance using screen-provided data. They were requested to cycle at a constant speed of between 15 and 20 km/h^1^. Simultaneously, speed and distance were recorded every 45 s. At the end of the cycling procedure, participants were informed their scores in math questions, the distance they covered, and they were asked to answer four more questions assessing if they were tired, if they liked the way they exercised, how many animals they saw, and if they repeated the motivational word. In this study, all participants performed the exercise in the forest.

The HR, SpO_2_, and self-assessment of lower limb fatigue and dyspnea were performed before and at the end of each exercise condition (VR, no-VR, SSE-VR, and 6MWT) for each patient.

### 2.3. Statistical analysis

A power of 86% and confidence interval of 95% were adopted, with an estimated value for a type I error of 5% for the sample size calculation in this study. A value for 14 patients was obtained. However, because this is a new exercise method, we recruited 20 patients. The Kolmogorov–Smirnov test was used to assess the normality of the distribution. Analysis of variance for repeated measurements on one factor (one group × four measurements × four parameters) with the independent variable of the exercise method (6MWT, VR, no-VR, and SSE-VR) and dependent variables as the control parameters (HR, SpO_2_, and Borg scales-CR-10). Tukey's *post-hoc* test was used to locate any differences between the means. Relationships between continuous variables were assessed using Pearson's R correlation coefficients. All statistical analyses were performed using the statistical package IBM SPSS 21 (SPSS Inc., Chicago, Illinois, USA). The level of significance was set at P<0.05. Data were presented as the mean ± standard deviation and percentage (%), where appropriate.

## 3. Results

The results of the characteristics of the four types of exercises are presented in Table 2.

**Table 2:** Exercise characteristics.

**6-minute walk test (6MWT)**		
Distance	km	0.6 ± 0.1
Speed	km/h	5.8 ± 0.9
Duration	min	6.0 ± /
**Exercise with virtual reality mask (VR)**		
Distance	km	2.9 ± 0.2
Speed	km/h	18.1 ± 0.8
Duration	min	10.0 ± /
**Exercise without virtual reality mask (no-VR)**		
Distance	km	2.8 ± 0.2
Speed	km/h	18.0 ± 0.8
Duration	min	10.0 ± /
**Self-selected exercise with virtual reality mask (SSE-VR)**		
Distance	km	6.5 ± 0.9
Speed	km/h	18.0 ± 1.0
Duration	min	21.8 ± 2.9

Data are expressed as mean ± standard deviation.

**Figure 2:**
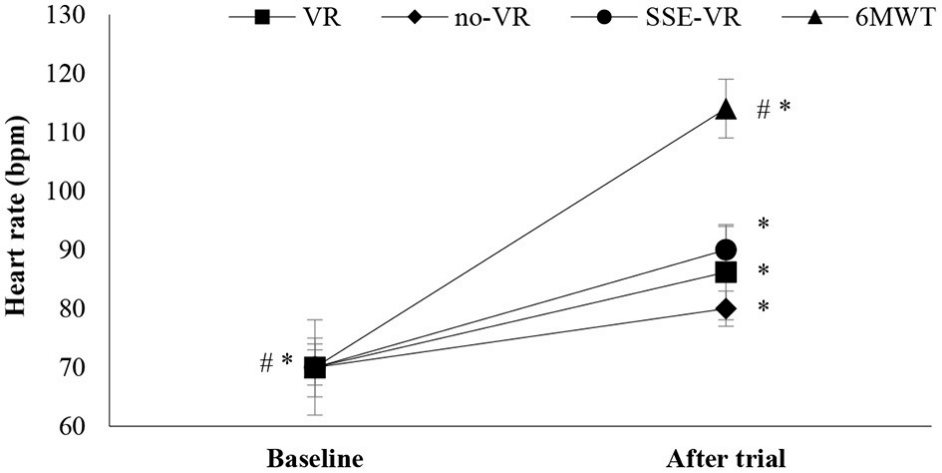
Heart rate changes between trials. *p < 0.001 between baseline and trials, ^#^*p* < 0.05 between trials [virtual reality (VR), exercise using the VR system; no-VR, exercise without using the VR system; SSE-VR, self-selected exercise using the VR system; 6MWT, 6-minute walk test].

The results showed differences between the four types of exercises in HR immediately after trials [*F*_(1.21, 22.97)_ = 26.25, *p* < 0.001, 6MWT: 113.9 ± 21.2 bpm; VR: 86.2 ± 8.9 bpm; no-VR: 83.8 ± 9.5 bpm; SSE-VR: 88.5 ± 9.8 bpm; Figure 2]. Higher values were observed in the 6MWT compare with those of exercise with VR at 21.9 ± 14.9%, no-VR at 23.9 ± 16.7%, and SSE-VR at 19.8 ± 15.7%. Self-selected exercise with VR showed higher values in HR immediately after trial compared with exercise with VR at 5.9 ± 4.4% and no-VR at 7.3 ± 9.8%. The results did not show differences between the VR and no-VR trials.

**Figure 3:**
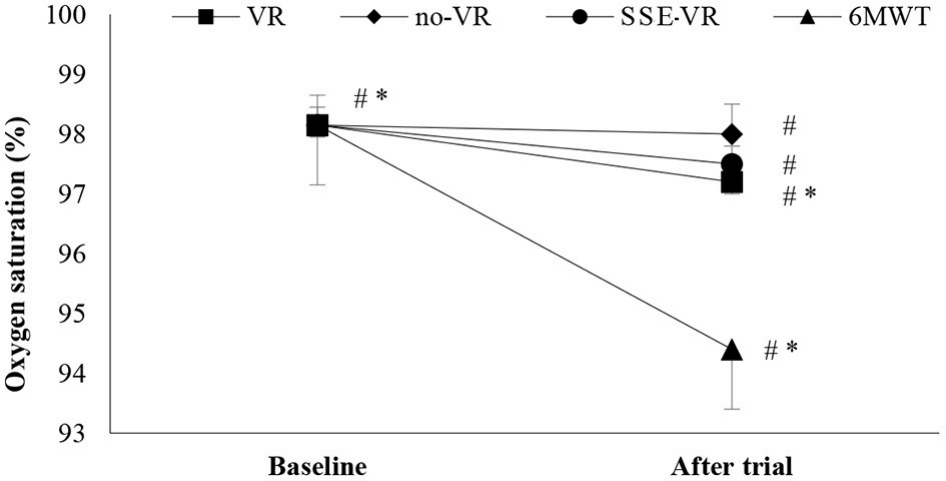
Oxygen saturation changes between trials. *p < 0.001, ^#^*p* < 0.05 [virtual reality (VR), exercise using the virtual reality system; no-VR, exercise without using the VR system; SSE-VR, self-selected exercise using the VR system; 6MWT, 6-min walk test].

The results showed differences between the four types of exercises in SpO_2_ immediately after trials [*F*_(1.17, 22.18)_ = 28.56, *p* < 0.001, 6MWT: 94.4 ± 2.6%; VR: 97.9 ± 0.9%; no-VR: 98.0 ± 0.8%; SSE-VR: 97.5 ± 1.1%; Figure 3]. The 6MWT observed higher desaturation compare to VR at 3.5 ± 2.5%, no-VR at 3.7 ± 2.8% and SSE-VR at 8.3 ± 6.7%. Self-selected exercise with VR showed lower values in oxygen saturation immediately after trial compared with exercise with VR at 13.0 ± 11.7% and no-VR at 13.2 ± 12.2%. The results did not show any differences between the VR and no-VR trials.

**Figure 4:**
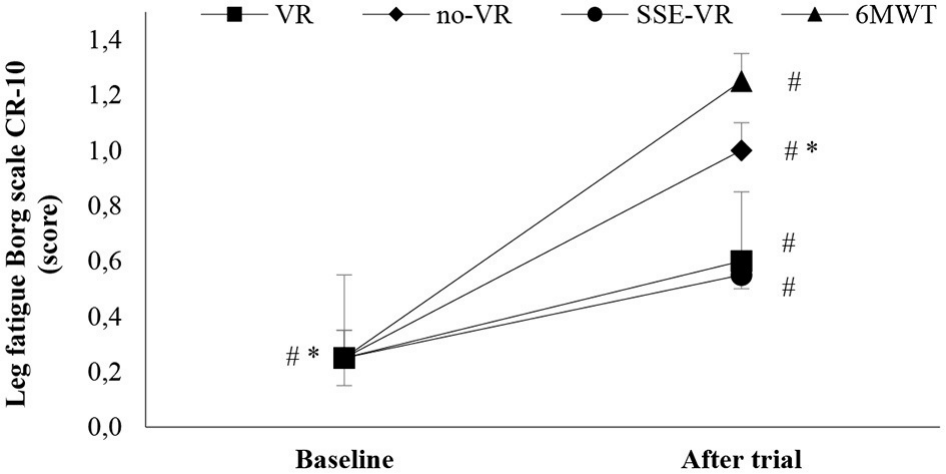
Leg fatigue changes between trials. *p < 0.001, ^#^*p* < 0.05 [virtual reality (VR), exercise using the virtual reality system; no-VR, exercise without using the VR system; SSE-VR, self-selected exercise using the VR system; 6MWT, 6-min walk test].

The results of self-reported leg fatigue immediately after the trials showed differences between the four types of exercises [*F*_(2.43, 46.11)_ = 3.82, *p* = 0.022, 6MWT: 1.3 ± 1.0; VR: 0.6 ± 0.9; no-VR: 0.7 ± 1.0; SSE-VR: 0.6 ± 0.7; Figure 4]. The 6MWT showed a higher score in leg fatigue than with exercise with VR at 73.3 ± 42.7%, no-VR at 64.2 ± 46.6%, and SSE-VR at 55.0 ± 51.0%. The results did not show a difference between the exercise conditions (VR, no-VR, and SSE-VR). The results did not show differences between the VR and no-VR trials.

**Figure 5:**
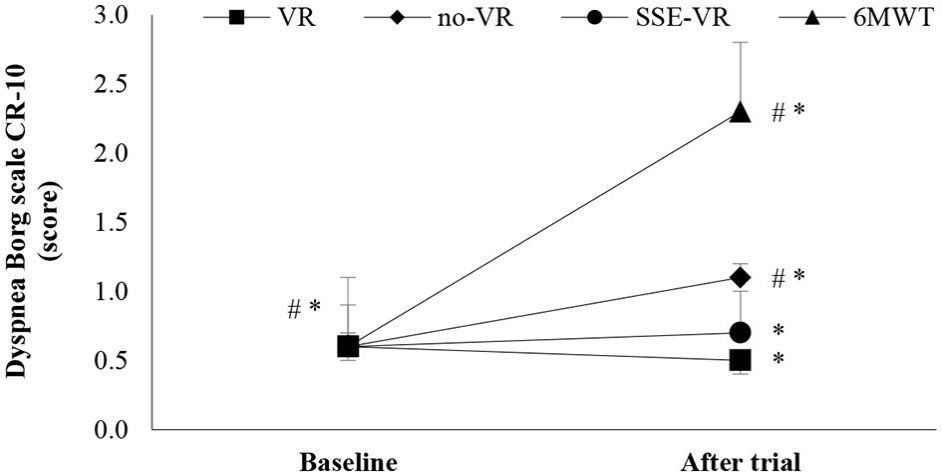
Dyspnea changes between trials. *p < 0.001, ^#^*p* < 0.05 [virtual reality (VR), exercise using the virtual reality system; no-VR, exercise without using the VR system; SSE-VR, self-selected exercise using the VR system; 6MWT, 6-min walk test].

The results of self-reported dyspnea immediately after the trials showed differences between the four types of exercises [*F*_(1.91, 36.25)_ = 15.08, *p* < 0.001, 6MWT: 2.3 ± 1.4; VR: 0.5 ± 0.6; no-VR: 1.1 ± 0.9; SSE-VR: 0.7 ± 0.7; Figure 5]. The 6MWT showed a higher score in dyspnea than with exercise with VR at 18.2 ± 40.5%, no-VR at 58.3 ± 28.9% and SSE-VR at 54.5 ± 47.2%. The results showed differences between the VR and no-VR trials [*t*_(19)_ = −3.269, *p* = 0.004].

The results in mean arterial pressure immediately after trials did not show differences between the four types of exercises (*p* > 0.05, 6MWT: 1,001.6 ± 8.4 mmHg; VR: 103.0 ± 9.4 mmHg; no-VR: 102.0 ± 8.4 mmHg; SSE-VR: 101.9 ± 8.1 mmHg). The results did not show differences between the VR and no-VR trials. A statistically significant correlation was detected between handgrip strength and leg fatigue at the end of the VR (*r* = −0.621, *p* = 0.003), SSE-VR trial (*r* = −0.450, *p* = 0.047), and dyspnea at the end of the SSE-VR trial (*r* = −0.484, *p* = 0.030). The WAI score was related to dyspnea at baseline (*r* = −0.450, *p* = 0.046) and dyspnea at the end of the VR trial (*r* = −0.508, *p* = 0.022). Body fat percentage (29.0 ± 5.1%) correlated to dyspnea at the end of the SSE-VR (*r* = 0.461, *p* = 0.041) and 6MWT (*r* = −0.498, *p* = 0.025). Percent of muscle mass (31.7 ± 2.5%) correlated to dyspnea at the end of SSE-VR trial (*r* = −0.466, *p* = 0.038).

## 4. Discussion

In our study, we investigated breathlessness in long-post-COVID-19 patients with mild cognitive impairment during exercise with and without using a VR system. In our analyses, dyspnea was associated with exercise type, body composition parameters, handgrip strength, and WAI score. Dyspnea was recorded to be lower during exercise with VR than during exercise without VR.

Previous studies have reported that the perception of breathlessness is related to disease severity, reduced chemosensitivity, age, sex, and reduced muscle force. In addition, psychological factors also play a role in breathlessness perception, and fear of hospitalization and loneliness may also contribute to breathlessness (28). In our study, the method exercise with the VR system showed lower scores for dyspnea and leg fatigue than the no-VR system. The neuropsychological processes underlying the perception of dyspnea pattern activation observed in the brain areas suggest a possible role for neural deficits in the blunted perception of dyspnea (29). Moreover, dyspnea activates a network of sensorimotor, cerebellar, limbic, and emotion-related areas (30). Exercise with VR systems is often used in neurorehabilitation therapies (patients with multiple sclerosis) and can lead to reduced levels of fatigue (31). In addition, exercising in a VR environment can lead to reduced feelings of pain and fatigue, such as in patients with spinal cord injury, compared with exercising in a no-VR environment (32).

Previous studies have shown that exercise with a VR system is interesting and enjoyable (15). The usefulness of exercising with a VR system compared with no VR conditions has also been reported to promote physical and cognitive health in patients with mild cognitive impairment. They found that the VR system was acceptable, tolerable, and usable by the patients (27). In our study, all patients answered the cognitive questions included in the experimental procedure correctly, irrespective of the condition they were exercising (VR or no-VR). Therefore, mild cognitive impairment had no effect on patient performance in our study.

Our study included patients who had been previously hospitalized for COVID-19 and had low oxygen saturation. The low PaO_2_ or SpO_2_ is primarily due to difficulty in maintaining normal PaO_2_ or SpO_2_ levels, resulting in cellular hypoxia (33), while the physiologically expected value depends on age and is approximated by the formula: PaO_2_ (mmHg) = 102–[0.33 × (age in years)]. Signals carried *via* the vagus nerve stimulate the central stem chemoreceptors. Increased CO_2_ in arterial blood lowers the pH (<7.4) and shifts the hemoglobin saturation curve to the right, thereby increasing dyspnea. Dyspnea was characterized by coughing during the 6MWT, which indicated a state of discomfort. The 6MWT is likely to cause activation of anaerobic metabolism due to low exercise capacity, leading to lactate production, while the mechanism of action for lactate neutralization is by plasma NaHCO_3_, resulting in the production of sodium lactate and the release of CO_2_, which in turn is incorporated into the CO_2_ carried in the blood. Hypercapnia increases pulmonary ventilation beyond the levels necessary for adequate blood oxygenation. Long-post-COVID-19 patients had arterial O_2_ saturation at rest at normal levels (98.2 ± 0.7%), while significant differences appeared after the 6MWT (94.4 ± 2.6%). The arteriovenous O_2_ difference depends on the rate of muscle uptake of blood-supplied O_2_ and is affected by changes in the oxyhemoglobin saturation curve. The decrease in venous blood O_2_ content is responsible for the increase in arteriovenous O_2_ difference and depends on the type, intensity, and duration of the exercise.

The unpleasantness of dyspnea is related to the activation of the limbic system, right anterior insula, and amygdala (28), while according to Hentsch et al. (28), unmyelinated C-fibers and small-diameter myelinated Aδ-fibers in the lungs and lower respiratory tract transmit mechanical and chemical signals to the nucleus tractus solitarius in the brain stem through the afferent vagal nerve. Sensory information from the lungs is transmitted to the brain through the vagus nerve. Sensory afferents in the vagus nerves are associated with dyspnea as they provide information about lung volume changes and permit awareness of the level of ventilation, which is an essential part of the sensation of dyspnea (34). Anxiety and depression can increase the intensity of dyspnea, and a patient's affective state can limit exercise tolerance. This may have resulted in the reduction of benefits of exercise on health. Researchers have found that an area in the right posterior cingulate cortex is activated during air hunger and is associated with affective cognitive factors (35). Negative emotional situations compared with positive emotional situations were associated with similar intensity levels of dyspnea but with a higher feeling of unpleasantness. Higher unpleasantness is associated with activation of the limbic system (anterior insula and amygdala) (36). Our results show that a single VR session aimed at promoting relaxation, distraction, and stress relief has beneficial effects on factors, such as tiredness, shortness of breath, anxiety, and depression compared with non-specific VR content. Thus, VR is considered a useful and safe tool that may help COVID-19 patients manage symptoms of tiredness, shortness of breath, and anxiety (37).

Moreover, breathlessness parallels the loss of elasticity in lung tissue, while lung stiffness also affects a patient's ability to expel CO_2_, and the build-up of this gas is a potent trigger for our urge to inhale (38). In our study, patients had elasticity of the chest Δchest >6 cm, and the indicator of spirometry peak expiratory flow, which is related to respiratory muscle strength, was 112.0 ± 11.3% of the predicted values. These scores were characterized as normal (21) and with good Δchest.

### 4.1. Implications for clinical practice

VR technology can extend the clinical applications of the overall management of patients, implementing existing cardiopulmonary rehabilitation programs in a pleasant environment (39). Such extensions could not be restricted to long-post-COVID-19 syndrome, but rather to several cardiorespiratory diseases, complying with the inclusion criteria (40). It is well-known that sedentary lifestyle is a crucial risk factor for cardiovascular and respiratory diseases. Patients struggle to incorporate exercise into their daily routines, especially when unguided. Rehabilitation aims to offer an organized program to familiarize patients with training. Telerehabilitation has gained popularity because of the convenience of guiding patients without forcing them to visit the laboratory (16, 41). VR is a novel approach that is easily distributed and utilized even in telerehabilitation/exercise (42–44). Participants found it pleasant and motivating to engage in exercise, maximizing their performance. According to our study findings, future research can be conducted to assess the impact of rehabilitation in patients with non-communicable diseases, which can be a future research scope of the current study.

### 4.2. Limitations of study

Despite the novel approach of our study, it is crucial to note some of its limitations. All participants were infected with the delta variant, which, in contrast to the omicron variant, exerted less neurotropism. Furthermore, sleep disturbances as a common comorbidity signif mild cognitive impairment, and therefore, proper cooperation in rehabilitation programs could be masked by such confounding factors, meaning that more studies are required to elucidate the causal interrelationship.

## 5. Conclusion

In conclusion, VR applications seem to be an attractive and safe tool for implementing rehabilitation. Their incorporation in rehabilitation programs could enhance performance during exercise and benefit patients with both reported respiratory and cognitive symptoms.

## Data availability statement

The original contributions presented in the study are included in the article/supplementary material, further inquiries can be directed to the corresponding author.

## Ethics statement

The studies involving human participants were reviewed and approved by approval number: 1829. The patients/participants provided their written informed consent to participate in this study.

## Author contributions

VTS, YT, and KIG conceived of the presented idea and designed the study. VTS and SB conceived of the patients recruitment. VTS, PK, KT, GT, DM, and EP contributed to sample collection, sample preparation, and data analysis. VTS, GDV, ET, MH, and KA contributed equally to the writing the paper. ZD, YT, and KIG supervised the study. All authors have read and agreed to the published version of the manuscript.
